# Location of the accessory infraorbital foramen with reference to external landmarks and its clinical implications

**DOI:** 10.1038/s41598-020-65330-4

**Published:** 2020-05-22

**Authors:** Kang-Jae Shin, Shin-Hyo Lee, Min-Gyu Park, Hyun Jin Shin, Andrew G. Lee

**Affiliations:** 1 Department of Anatomy and Cell Biology, Dong-A University College of Medicine, Busan, Republic of Korea; 2 Department of Anatomy, Yonsei University College of Medicine, Seoul, Republic of Korea; 3 Department of Oral Anatomy, School of Dentistry, Pusan National University, Pusan, Republic of Korea; 4 Department of Ophthalmology, Research Institute of Medical Science, Konkuk University Medical Center, Konkuk University School of Medicine, Seoul, Republic of Korea; 5 Department of Ophthalmology, Blanton Eye Institute, Houston Methodist Hospital, Houston, Texas USA; 6 Department of Ophthalmology, Neurology, Neurosurgery, Weill Cornell Medicine, New York, NY USA; 7 Department of Ophthalmology, University of Texas Medical Branch, Galveston, Texas USA; 8 Department of Ophthalmology, UT MD Anderson Cancer Center, Houston, Texas USA; 9 Department of Ophthalmology, Texas A and M College of Medicine, College Station, Texas USA; 10 Department of Ophthalmology, University of Iowa Hospitals and Clinics, Iowa City, Iowa USA; 11 Department of Ophthalmology, Baylor College of Medicine and the Center for Space Medicine, Houston, TX USA; 12 Department of Ophthalmology, University of Buffalo, Buffalo, New York USA

**Keywords:** Somatic system, Visual system

## Abstract

The aim of this study was to define the location of the accessory infraorbital foramen (AIOF) with reference to accessible external landmarks in order to facilitate orbital and oculoplastic surgical procedures in the maxillofacial region. Forty-four hemifaces from 25 cadavers were dissected. The lateral canthus, subnasal point, and lacrimal caruncle were used as anatomic reference points. The AIOF was observed in 8 of the 44 hemifaces (18.2%) and was situated at a mean distance of 7.2 mm superomedial to the IOF. The horizontal distance from the lacrimal caruncle to the AIOF was 0.3 mm. In all cases the AIOF was situated at a point that was no more than 8 mm from the intersection point of a vertical line passing through the lacrimal caruncle and an oblique line joining the lateral canthus and the subnasal point. Surgeons anesthetizing or performing surgical procedures in the maxillofacial region should be aware of the frequency of the AIOF (18.2%) and its location (on the superomedial side of the IOF). We propose that injecting at the intersection point of a vertical line passing through the lacrimal caruncle and an oblique line joining the lateral canthus and the subnasal point would successfully block the accessory branch of the infraorbital nerve. Likewise, surgeons operating in this region should be aware of the location of the AIOF in order to avoid inadvertent iatrogenic injury to a duplicated infraorbital nerve.

## Introduction

The infraorbital nerve (ION) derives from the trigeminal nerve exiting the maxilla via the infraorbital foramen (IOF) and provides midface sensation^1^. Regional nerve block of the ION can be used for regional anesthesia during surgeries involving the midface region or when treating neuralgia of the infraorbital nerve with a complaint of unusual facial pain. However, sometimes the ION is duplicated, and the presence of the accessory infraorbital nerve (AION) often results in anesthesia failure^2,3^. Thus, clinicians should consider the presence of this accessory branch passing through accessory infraorbital foramen (AIOF) in order to accomplish satisfactory anesthesia of the midfacial region and also to avoid iatrogenic injury to the duplicated ION.

While the ION has been investigated extensively in the prior literature, there are few reports on the topography of the AIOF, resulting in insufficient information on this foramen in the literature^4–6^. Previous studies determining the location of the AIOF used the nasomaxillary suture and anterior nasal spine as reference landmarks^7,8^. However, palpitating such deep bony landmarks externally is very difficult, and so they are rarely used in clinical situations, which makes it necessary to identify identifiable external landmarks as references for easily predicting the location of the AIOF.

Previously we have reported the usefulness of the lateral canthus^9,10^, lacrimal caruncle^11,12^, and subnasal point^13^ as external anatomic landmarks in oculofacial surgery. These landmarks are accessible and static, and so could be suitable points of reference for identifying deeper structures such as nerves and arteries when attempting to prevent iatrogenic injuries and facilitate dissections. This study used cadaveric dissection with the purpose of determining whether these reference points could serve as reliable landmarks for identifying the underlying AIOF. Such information may assist clinicians to perform safer and more effective surgical procedures as well as in local anesthesia planning.

## Materials and Methods

Fifty hemifaces of 25 embalmed adult Korean cadavers legally donated in the Dong-A University College of Medicine were included in this study. After excluding six hemifaces that had been anatomically disrupted by previous surgery, trauma or prior dissections in the midface region, 44 hemifaces were suitable for morphometric measurements (comprising 19 male and 6 female orbits, and 22 right and 22 left hemifaces) and examined independently of their right and left halves. The mean age at the time of death was 73.7 years (range, 44–96 years). All procedures were performed in accordance with the Declaration of Helsinki of the World Medical Association (WMA). After the approval of the Dong-A University College of Medicine, twenty-five embalmed cadavers that were legally donated to this institution, were subjected to the dissection of the maxillofacial region. The cadavers were not from prisoners and that informed consent was obtained from the legal guardians.

### Measured parameters

**Figure 1:**
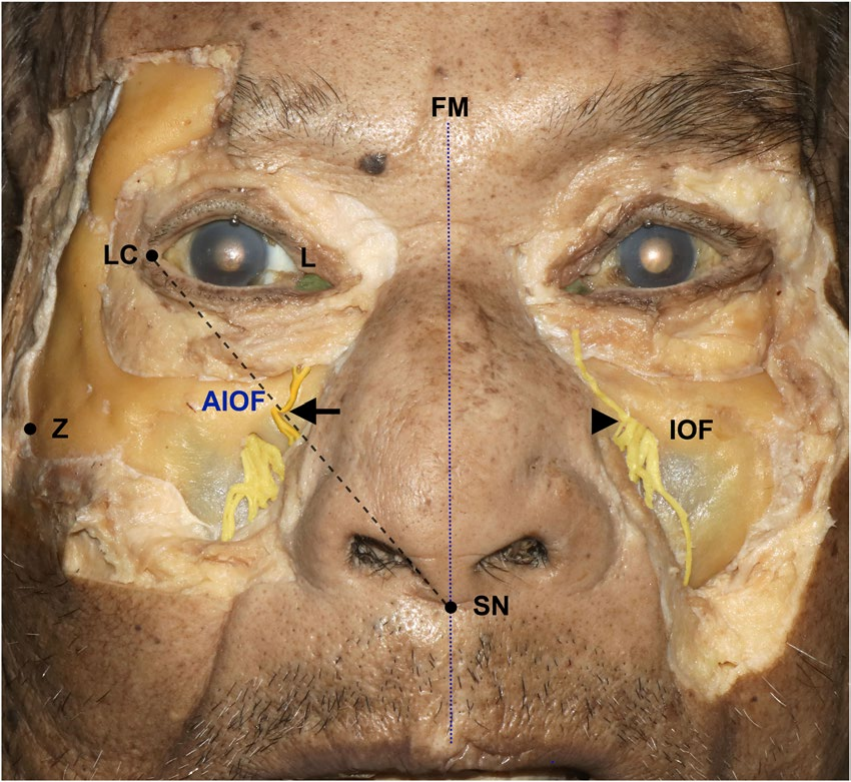
Cadaveric dissection demonstrating the external landmarks. The oblique line joining the lateral canthus (LC) and the subnasal point (SN) passed through the accessory infraorbital foramen (AIOF). The arrow and arrowhead indicate the AIOF and infraorbital foramen (IOF), respectively; FM, facial midline; L, lacrimal caruncle; Z, zygion.

Each cadaver was placed in a supine position, and the skin and subcutaneous tissue over the maxilla were cut and removed completely. The location of the AIOF was determined using the following external landmarks: lateral canthus, subnasal point, and the lacrimal caruncle (Fig. 1). We measured the following parameters directly on cadavers using digital calipers:

#1.Horizontal distance from the facial midline to the lacrimal caruncle.

#2.Horizontal distance from the facial midline to the lateral canthus.

#3.Horizontal distance from the facial midline to the zygion.

#4.Vertical distance from the AIOF to the lacrimal caruncle.

#5.Vertical distance from the AIOF to the infraorbital margin.

#6.Horizontal distance from the AIOF to the lacrimal caruncle.

#7.Horizontal distance from the AIOF to the facial midline.

#8.Distance from the AIOF to the IOF.

#9.Angle between the AIOF and the horizontal line through the IOF.

### Statistical analysis

Statistical analyses were performed using SPSS (version 18.0, SPSS, Chicago, IL, USA). We used the Shapiro-Wilk test for determining whether the data conformed to a parametric or nonparametric distribution. Full faces were examined in their two halves in the 19 cadavers and the side difference (laterality) was verified using Paired samples-t test. The linear relationships between the study parameters (between #3 and #2 and between #3 and #7) were calculated using Pearson’s correlation test. Conclusions are made at *p* = 0.05 level of significance.

## Results

**Figure 2:**
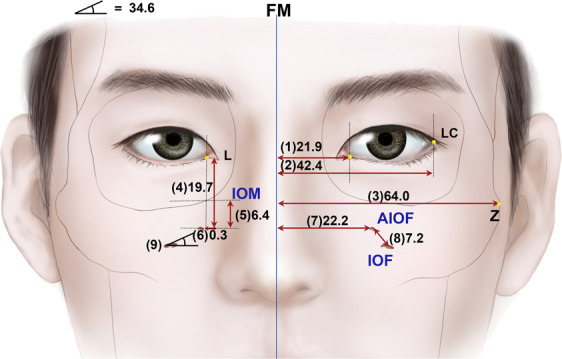
Illustration of the main vertical and horizontal parameters; IOM, infraorbital margin. Measurement unit (millimeter), except #9 (degree).

All of the study parameters were measured twice by two of the authors (K.J.S. and S.H.L). The intergrader reliability for these two graders was confirmed by the к value ranging from 0.90 to 0.94 for different variables. All of the measurements conformed to a normal distribution. There was no laterality difference in the variables between the left and right sides of the hemiface. The values of parameters are listed in Table 1 and displayed in Fig. 2.

**Table 1:** Measured parameters. Detailed descriptions are provided in the main text and Fig. 2.

Anatomic parameter		#	Mean	SD
Horizontal distance from facial midline	to lacrimal caruncle	(1)	21.9	1.7
	to lateral canthus	(2)	42.4	2.2
	to zygion	(3)	64.0	4.9
Vertical distance from AIOF	to lacrimal caruncle	(4)	19.7	1.9
	to infraorbital margin	(5)	6.4	1.4
Horizontal distance from AIOF	to lacrimal caruncle	(6)	0.3	3.5
	to facial midline	(7)	22.2	2.9
Distance from AIOF	to IOF	(8)	7.2	2.4
Angle between horizontal line through IOF	and AIOF	(9)	34.6	14.9

#, number of measured parameter

Values are in millimeters, except for #9 (in degrees)

IOF, infraorbital foramen; AIOF, accessory infraorbital foramen.

The dimensions related to AION from the lacrimal caruncle—the vertical (#4) and horizontal (#6) distances—had values of 19.7 ± 1.7 mm (mean ± SD) and 0.3 ± 3.5 mm, respectively. The horizontal distance related to the AIOF from the facial midline (#7) had a value of 22.2 ± 2.9 mm. Pearson’s correlation tests between the distances (#3 and #2, and #7) were analyzed. Distance #3 was positively associated with #2 and #7 (both *p* < 0.05). According to the increase in the horizontal lengths on the midface (#2), the lateral canthus (#2) and AIOF (#7) tended to be located more laterally.

**Figure 3:**
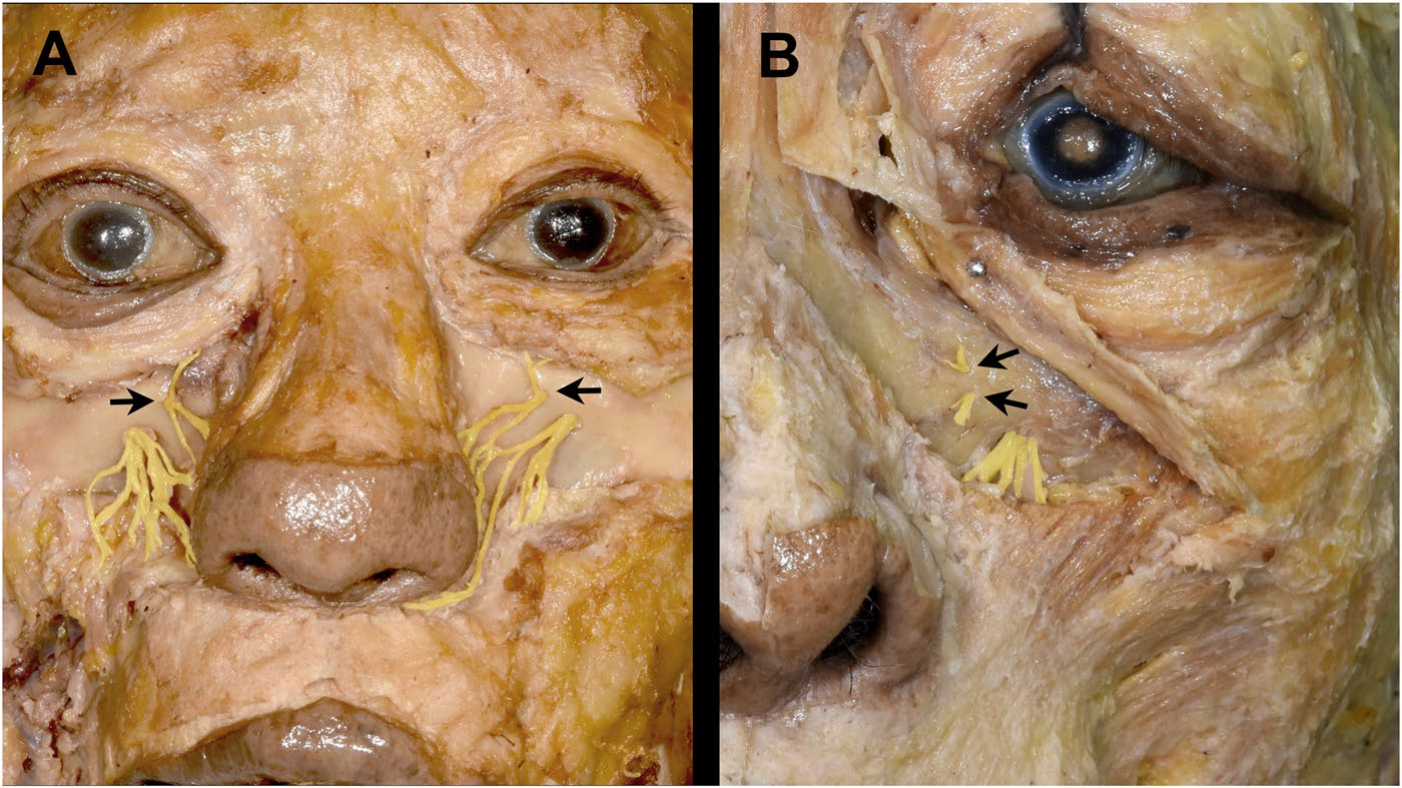
Cadaveric dissection showing the anatomic and morphometric variations of the AIOF. **(A)** The AIOF occurred bilaterally in two specimens (arrows, 25%). **(B)** Double accessory foramina were present in one specimen (arrows, 12.5%).

**Figure 4:**
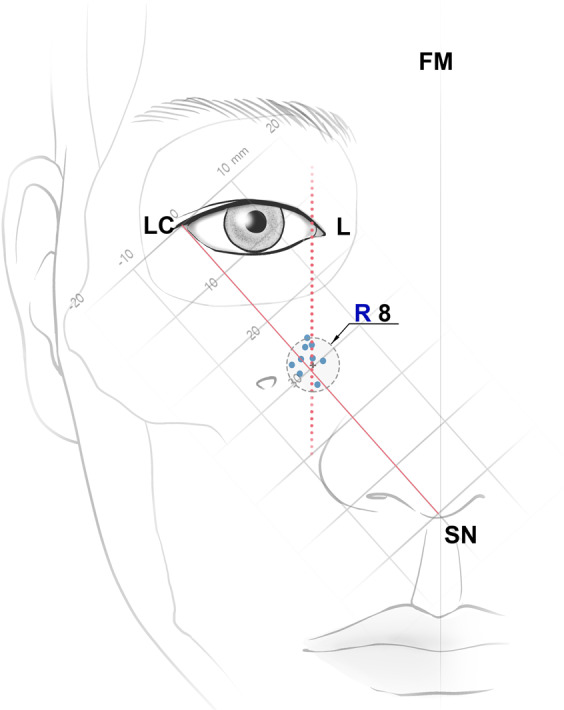
Schematic drawing of the distribution of the AIOF. All of the accessory foramina were located within 8 mm of the intersection point between the vertical line passing through the L and the oblique line joining the LC and the SN; R, radius. Unit: millimeters.

The AIOF was observed in 8 of the 44 hemifaces (18.2%), with 5 and 3 cases located on the right and left sides, respectively, while 2 cases were bilateral (Fig. 3A). There was one case in which two holes were found on one side (Fig. 3B). Figure 4 displays the distribution of the AIOF in the eight cases: it was located superomedially to the IOF in 88.9% and medially in 11.1%. In most cases the AION was situated where a vertical line passing through the lacrimal caruncle intersected with an oblique line joining the lateral canthus and the subnasal point. The distance from the point of intersection to the AIOF ranged from 4 to 8 mm.

## Discussion

Locating the ION can be difficult and the presence of a double foramina is important when planning surgery and/or local anesthesia in the maxillofacial region. When a clinician performs ION block, nerve blocks may not provide adequate analgesia if an AION is present. It is obvious that the presence of extra branches of the nerve can result in possible iatrogenic morbidity during facial surgery, and so a surgeon should be aware of and consider this anatomic variation.

**Figure 5:**
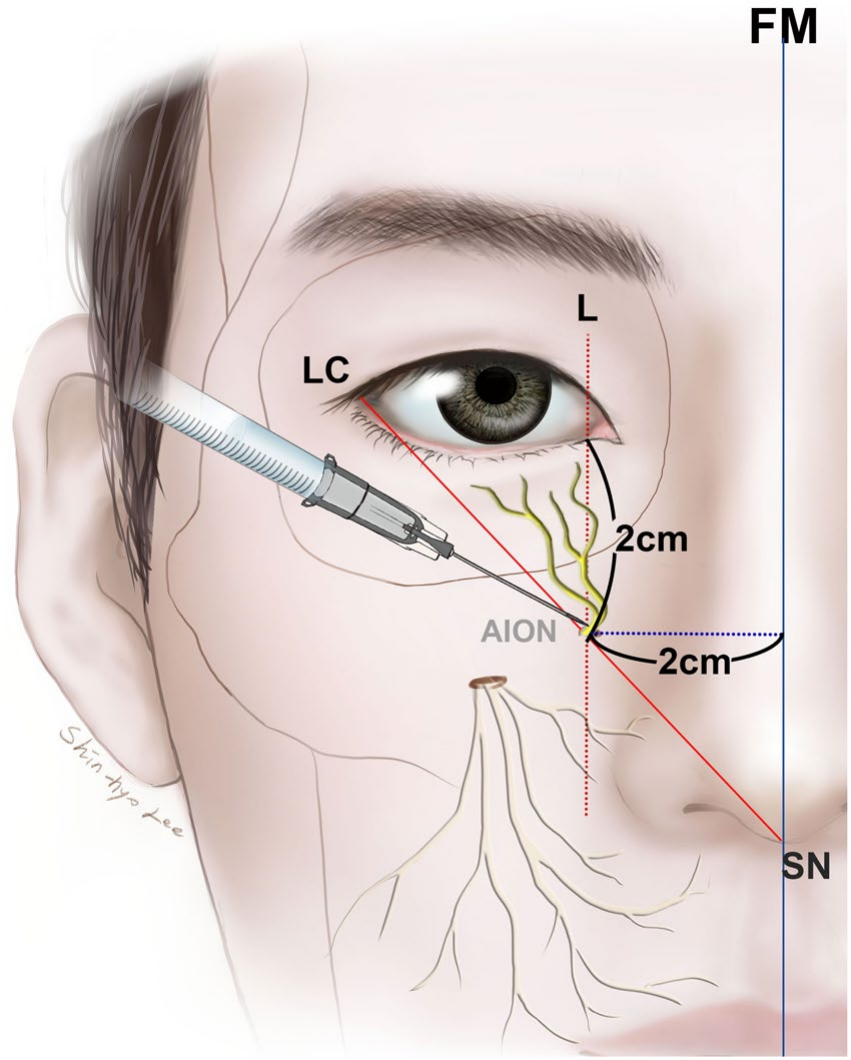
Guideline for the injection site of anesthetic for achieving successful AION block during surgeries involving the maxillofacial region. The optimum site for anesthetic injections is the intersection point between the vertical line passing through the L and the oblique line joining the LC and the SN. Also, the point located 2 cm below the lacrimal caruncle and 2 cm lateral from the facial midline could be consider as alternative injection site.

The present study has revealed the topography of the AIOF in Korean cadavers. The AIOF was found in 18.2% of cases and was mostly located superomedial to the IOF with a single accessory foramen. We also found that in all cases the AIOF was located within 8 mm of the point where a vertical line passing through the lacrimal caruncle intersected with an oblique line joining the lateral canthus and the subnasal point. Considering how anesthetic agent is distributed, injecting into this intersection point would successfully block the AION in most patients (Fig. 5). Alternative landmarks could be used if facial trauma or another ocular anomaly has changed the original positions of these soft-tissue landmarks. The vertical and horizontal distances from the AIOF to the lacrimal caruncle and the facial midline were 19.7 mm (#4) and 22.2 mm (#7), respectively. Thus, it can also be concluded that the AIOF is situated approximately 2 cm below the lacrimal caruncle and 2 cm lateral from the facial midline (Fig. 5).

**Figure 6:**
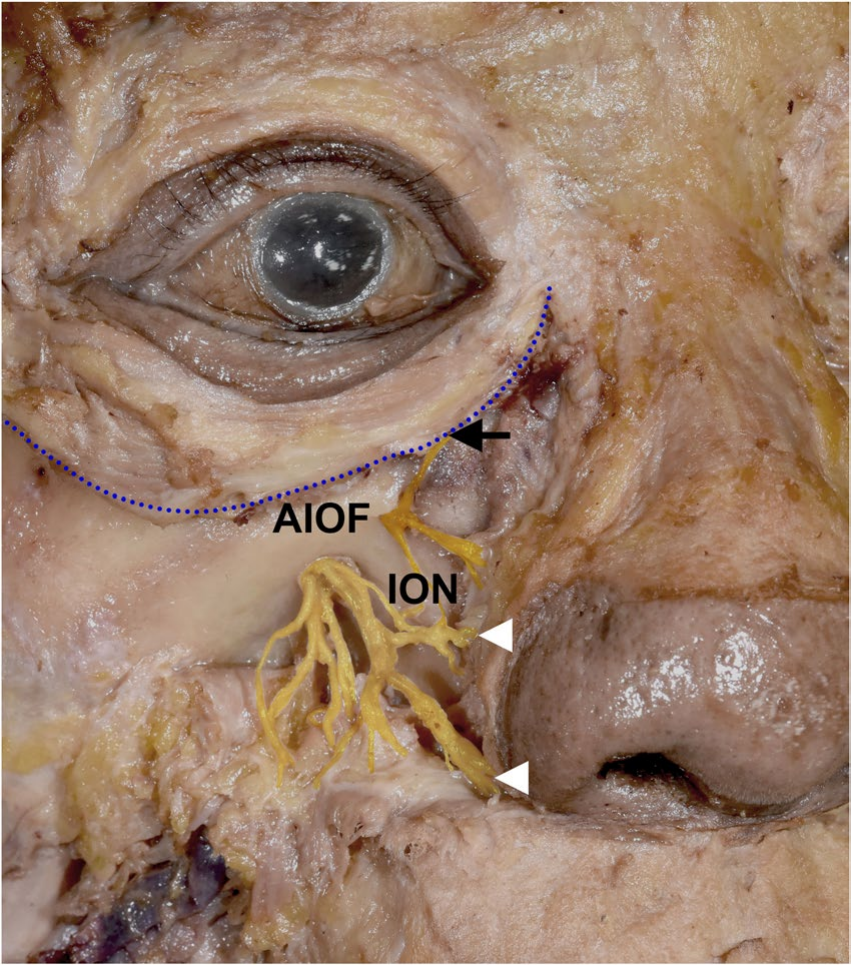
Sensory innervation of lower eyelid when an AIOF is present. The lower eyelid always receives sensory innervation from the accessory branch of the infraorbital nerve (arrow) instead of the ION (arrowhead), a trend that can also be observed in Figs. 1 and 3.

One particularly interestingly finding was that the presence of an AION will result in the lower eyelid always receiving sensory innervation from that accessory nerve instead of the ION (Fig. 6). In some cases, inadequate ION block by local anesthesia when performing lower eyelid surgery may therefore be associated with this anatomic variation, and so if the patient still feels pain even after performing an ION block, the surgeon should consider an additional injection into the above-mentioned intersection point in order to achieve a sufficient anesthetic effect.

Several studies have documented the location of the AION with reference to several deep bony landmarks such as the frontomaxillary suture, zygomaticomaxillary suture, and anterior nasal spine^7,8^. Although predicting the location of the AIOF using these anatomical landmarks has been described previously, most of these reports were confined to the research laboratory setting and are too complex for use in the clinic or operating room. Also, these structures are somewhat distant from the AIOF; for example, the frontomaxillary suture has been documented to be approximately 25 mm from the AIOF^8^. In addition, most of the previous studies only examined dry skulls, and not cadavers, and it cannot be confirmed whether or not the nerve passes through the foramen in the dry skull. For example, there is a report of an AIOF frequency of 47.6% in the dry skull, which is much higher than the commonly reporting prevalence of 16.9%^14^.

One strength of our study is that—in order to make accurate assessments—we used embalmed cadavers instead of using dry skulls, and confirmed that nerves actually pass through the foramen. Little holes without nerves were not considered foramina in this study. Also, we used external landmarks that are more practical due to them being easily visible and palpable in the clinical situation. The soft-tissue landmarks used in the present study (e.g., lacrimal caruncle, lateral canthus, and subnasal points) have been demonstrated to be reliable anatomical landmarks for identifying underlying structures in previous studies, and appropriate descriptions have appeared for their utilization in all surgical disciplines^9–13^.

We analyzed the positional relationships between the AIOF and multiple external landmarks instead of measuring the distance of the AIOF from a single reference point. We adopted this approach since simply measuring a single distance does not reflect positional changes related to changes in the size of the face. The lateral canthus on the face is situated more laterally in larger skulls^9,15,16^. In the present study we found that in horizontally larger midfaces, the AIOF tend to be situated more laterally as well. This finding suggests that the lateral canthus is a reliable external landmark that will not be significantly influenced by individual differences in skull size. Our clinical observations revealed the AIOF to be situated around the oblique line joining the lateral canthus and the subnasal point, which is similar to the report of Ercikti *et al*.^17^. We also found that horizontally, the AIOF (#6) was situated 0.3 mm lateral from the lacrimal caruncle. Based on these findings, the intersection point of two lines—the oblique line joining the lateral canthus and the subnasal point and the vertical line passing through the lacrimal caruncle—could be ideal for identifying the location of the underlying AIOF.

With regard to the location of the AIOF, the findings made in this study are also similar to previous reports. Canan *et al*.^18^ reported that the AIOF was located 79.6% superomedial to the IOF, as similarly documented by Boopathi *et al*.^19^ Tezer *et al*.^7^ found that the AIOF was located superomedial to the IOF in 93.3% of cases and inferomedially in the other 6.7%; we found that 88.9% of them were located superomedially while the other 11.1% were located medially. For localization of the foramen, the reported distance from the AIOF to the IOF has varied from 3.95 to 9.98 mm^7,8^. The data obtained in our study were consistent with this, since the mean distance (#8) was 7.2 mm.

Clinicians can also consider an alternative anesthetic technique named the maxillary nerve block to address the presence of the AION. This technique involves a more-proximal approach at the level of maxillary nerve before it enters the infraorbital canal, and can be performed via a high tuberosity, greater palatine canal, and coronoid approach. Even if this method is more difficult to perform, it has the advantage of anesthetizing the maxillary nerve in the pterygopalatine fossa and providing profound analgesia for midface surgery regardless of whether the ION is duplicated. Also, real-time ultrasound-guided ION blocks could be another option. Considering individual variations in craniofacial morphology, an ultrasound-guided technique can help the physician to identify the underlying IOF and AIOF in individual patients and so also reduce the risk of direct puncturing the neurovascular bundle^20^. However, considering the possibility of a lack of ultrasound equipment and appropriately trained physicians, our suggested simple method would provide a convenient guideline and aid the clinician in achieving successful regional nerve block in the midface area.

This study had several limitations. First, the number of specimens was too small to determine the frequency of the presence of the AIOF in the Asian population. Achieving this would require calculation of the required sample size based on the total Asian population. Second, our dissections were performed only in Asian cadavers. Considering the anatomical variations in craniofacial bone among races, the results could differ in Caucasians and other races. Also, the frequency of the skull having an AIOF has reportedly varied according to the latitude^21^. Lastly, because of craniofacial growth and bony structural changes with age^22^, the topography of the AIOF as mainly obtained in elderly subjects in our study could be different in young subjects. Therefore, future studies with large samples including different age groups should investigate the frequency in particular regions of the world as well as racial differences.

In conclusion, considering our findings and previous reports, the relatively high prevalence of the AIOF means that a surgeon should be aware of this anatomic variation when performing any surgical or anesthetic procedures in the maxillofacial region. The intersection point of the vertical line passing though the lacrimal caruncle and the oblique line joining the lateral canthus and subnasal point can be used to accurately locate the AIOF. This new method of localizing the AIOF can assist clinicians performing AION blockade and thereby prevent iatrogenic AION damage, and also improve patient satisfaction.

## Data Availability

The datasets generated and analyzed during the current study are available from the corresponding author on reasonable request.
